# Mind the gap! Connexins and pannexins in physiology, pharmacology and disease

**DOI:** 10.3389/fphar.2013.00144

**Published:** 2013-11-21

**Authors:** Aida Salameh, Katja Blanke, Stefan Dhein

**Affiliations:** ^1^Department of Paediatric Cardiology, Heart Centre University of LeipzigLeipzig, Germany; ^2^Department of Cardiac Surgery, Heart Centre University of LeipzigLeipzig, Germany

**Keywords:** connexin, pannexin, mutation, stem cells, hemichannels, arrhythmia, inborn heart disease, cancer

Among other aspects it is the communication which makes the difference between a crowd of individuals and a society. Similarly, a key feature of an organism or of organs is the communication between their individual cells realized by mediators, hormones, and by direct intercellular communication via gap junction channels allowing the transmission of electrical signals and the exchange of small molecules to regulate growth and differentiation. This enables the organ or the organism to adapt very efficiently to the actual needs. Due to the important role of gap junction intercellular communication (GJIC) for the correct functioning of organs, and tissues a tight regulation of the expression of gap junction channel proteins, the connexins, their localization, and function is required. Besides connexins, another group of proteins, the pannexins, showing many molecular similarities with connexins have been identified. They seem to form hemichannels which may regulate cytosolic homeostasis or the release of small molecules. The present issue provides a comprehensive picture of recent developments and current research in this fascinating, fast developing area comprising review and original research articles on both connexins and pannexins written by leading experts in their research areas. The articles are organized in three parts:

- role of gap junctions in cell biology; regulation and targeting of connexins (11 articles)
- connexins and pannexins (3 articles)
- gap junctions in various diseases (6 articles)

Regarding part A, regulation of connexin function is not only realized via regulation of expression but also by various post-translational modifications as reviewed by Axelsen et al. (2013). Verheule and Kaese (2013) shed light on the different roles of cardiac connexins for cardiac phenotypes in various knock-out models. Connexins not only form intercellular dodecameric channels but also may form unopposed hemichannels, which may allow cAMP release as a new pathway for intercellular cAMP signaling as shown by Valiunas (2013). With regard to their role in differentiation and growth Oyamada et al. (2013) review the role of GJIC and connexins in development and re-programming of embryonic stem cells and induced pluripotent stem cells in comparison to the undifferentiated state. According to recent findings connexins not only have functions in the membrane, but also may control gene expression, and -as found by Boengler et al. (2013)-are expressed in the mitochondria where they control mitochondrial K^+^-influx. Another puzzling aspect of gap junction research is the ability or non-ability of certain connexins to form heteromeric channels which are composed of more than only one isoform. Regarding this aspect Beyer et al. (2013a) investigate the heteromeric interactions between Cx40 and Cx43 focusing on the role of the N-terminal. Regarding growth control by GJIC Kardami's group investigated the inhibition of DNA synthesis by Cx43 and S262-Cx43 de-phosphorylation Jeyaraman et al. (2013). In the next two articles histone-deacetylase is examined as a possible pharamcological target for influencing Cx43 expression with reduced expression when using trichostatin A (Xu et al., 2013) or with enhanced expression when using 4-phenylbutyrate (Kaufman et al., 2013). The section is closed with two more methodological articles showing a new cell culture system for the study of Cx29 (Söhl et al., 2013) and a new Escherichia coli expression system for Cx45 carboxyl terminus allowing the yield of large protein amounts as needed for NMR analysis (Kopanic et al., 2013).

Part B starts with an original article about pannexin 1 elucidating the problems with Panx1 knock outs and showing the generation of astrocyte and neuron-specific Panx1 deletions (Hanstein et al., 2013). The role of Panx1 and connexin hemichannels in brain glial cells in health and disease as well their impact for neuroglial interaction and possible pharmacological approaches are reviewed by Giaume et al. (2013). The distribution of Panx1 in four rat brain regions using different antibodies in a comparative study is investigated by Cone et al. (2013).

Part C focuses on the role of gap junctions in various diseases starting with an interesting hypothesis article by Végh et al. (2013) on the regulation of cardiac gap junctions by nitric oxide in ischemia and reperfusion and its relation to arrhythmia. In the next article the inverse relationship between proliferative activity of a tumor induced by cardiac transplantation of bone marrow stem cells and intra-tumor connexin expression. Spath et al. (2013) conclude that the lack of connexin expression in the most proliferative areas of the tumor results in absence of differentiation and growth stop signals so that invasive growth is facilitated. The role of gap junction mutations or alteration in inborn human heart disease is reviewed by Salameh et al. (2013) in comparison to the findings in various mouse models. Beyer et al. (2013b) review the role of connexin mutations in the pathogenesis of lens cataracts and discuss altered hemichannel functions and formation of cytoplasmic accumulations. Interesting new aspects about a possible pathogenetic role of gap junctions are given by Blanke et al. (2013) with regard to the formation of infantile hemangiomas and possible interference between beta-adrenoceptors and connexins. The section closes with a review on the most recent advances in research on GJIC and oculodentoglial dysplasia focussing on the possible relationship between channel dysfunction and neurological symptoms in these patients De Bock et al. (2013).

This compilation of articles on most recent developments in connexin and pannexin research hopefully encourages more scientists to investigate these highly interesting cell biology mechanisms in their research areas. When cellular interaction or interplay is of relevance this recent research suggests: mid the gap!
